# Psychometric Properties of a Short Self-Reported Measure of Medication Adherence Among Patients With Hypertension Treated in a Busy Clinical Setting in Korea

**DOI:** 10.2188/jea.JE20130064

**Published:** 2014-03-05

**Authors:** Jeung-Hee Kim, Weon-Young Lee, Yeon-Pyo Hong, Wang-Seong Ryu, Kwang Je Lee, Wang-Soo Lee, Donald E. Morisky

**Affiliations:** 1 Division of Chronic Disease Control, Korea Centers for Disease Control and Prevention, Osong, Republic of Korea; 2 Department of Preventive Medicine, College of Medicine, Chung-Ang University, Seoul, Republic of Korea; 3 Department of Internal Medicine, Seoul Circulation Clinic, Seoul, Republic of Korea; 4 Division of Cardiology, Department of Internal Medicine, Chung-Ang University Hospital, Seoul, Republic of Korea; 5 Department of Community Health Sciences, Fielding School of Public Health, University of California at Los Angeles, Los Angeles, CA, USA

**Keywords:** medication adherence, self-report, questionnaires, psychometrics, hypertension

## Abstract

**Background:**

We examined the psychometric properties of the Korean version of the 8-item Morisky Medication Adherence Scale (MMAS-8) among adults with hypertension.

**Methods:**

A total of 373 adults with hypertension were given face-to-face interviews in 2 cardiology clinics at 2 large teaching hospitals in Seoul, South Korea. Blood pressure was measured twice, and medical records were reviewed. About one-third of the participants (*n* = 109) were randomly selected for a 2-week test-retest evaluation of reliability via telephone interview.

**Results:**

Internal consistency reliability was moderate (Cronbach α = 0.56), and test-retest reliability was excellent (intraclass correlation = 0.91; *P* < 0.001), although a ceiling effect was detected. The correlation of MMAS-8 scores with scores for the original 4-item scale indicated that convergent validity was good (*r* = 0.92; *P* < 0.01). A low MMAS-8 score was significantly associated with poor blood pressure control (χ^2^ = 29.86; *P* < 0.001; adjusted odds ratio = 5.08; 95% CI, 2.56–10.08). Using a cut-off point of 6, sensitivity and specificity were 64.3% and 72.9%, respectively. Exploratory factor analysis identified 3 dimensions of the scale, with poor fit for the 1-dimensional construct using confirmatory factory analysis.

**Conclusions:**

The MMAS-8 had satisfactory reliability and validity and thus might be suitable for assessment and counseling regarding medication adherence among adults with hypertension in a busy clinical setting in Korea.

## INTRODUCTION

Inadequate adherence to antihypertensive drug therapy is a very common factor in uncontrolled blood pressure (BP).^1^^,^^2^ The World Health Organization (2003) estimated that adherence rates range from 50% to 70%, although the relevant studies varied with respect to study population, duration of follow up, and method used to assess adherence.^3^ In Korea, the rate of adherence to antihypertensive medications, as measured by self-report of regular medication use, was 61.1%.^4^ In the context of doctor-patient interaction in ordinary Korean clinical settings, this finding can be explained by 2 types of factors. First, many individuals with hypertension have negative feelings toward antihypertensive drugs and lack knowledge of hypertension. For example, they sometimes do not appreciate the need to continue medication even when they no longer have symptoms.^5^ Second, although physicians should carefully assess noncompliance with antihypertensive drugs and inform patients about hypertension management, they are unable to do so due to the limited time for physician consultation and lack of attention to BP control.^6^ These limitations might be due to characteristics of the Korean health care system, such as the fact that reimbursement for outpatients is based on a fee-for-service model rather than on capitation payment.

In this context, the simplicity of the 8-item Morisky Medication Adherence Scale (MMAS-8) as a self-reported measure could make it a very practical approach to assess adherence to an antihypertensive treatment regimen.^7^^,^^8^ Moreover, this questionnaire could help to stimulate physician-patient dialogue on antihypertensive medication because the scale items illustrate adherence behaviors that occur most frequently in practice.^8^ Although a self-report measure might be limited by recall bias and overestimation,^9^ the simplicity of such an instrument could reduce barriers to medication adherence and increase feasibility in busy clinics, thus offsetting those disadvantages.

We assessed the psychometric properties of the Korean version of the MMAS-8. The English version of the MMAS-8 has shown good validity and reliability among a population made up primarily of low-income black and Hispanic adults with hypertension in the United States.^10^ The reliability and validity of the MMAS-8 has also been examined among patients with diabetes in Thailand and Malaysia,^11^^,^^12^ among patients with hypertension in France,^13^ and among patients taking warfarin in Singapore.^14^

## METHODS

### Participants

**Figure 1.**
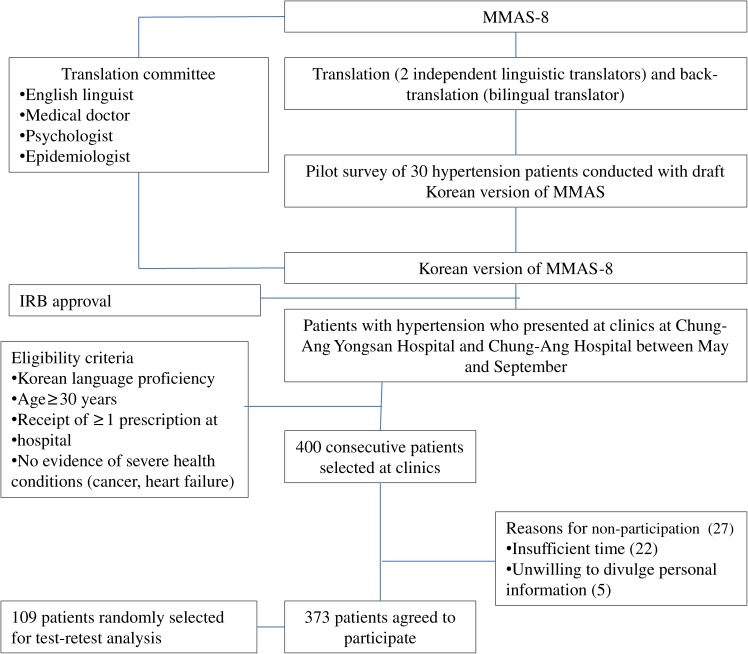
Study design. MMAS-8 = 8-item Morisky Medication Adherence Scale, IRB = institutional review board.

This study was performed between May and September in 2010 in cardiology clinics at the Chung-Ang University Yong San Hospital and Chung-Ang University Hospital in Seoul, which are large teaching hospitals in the same university educational foundation. To provide good precision for factor analysis, the estimated target sample size was 160 patients, using a ratio (sample size: number of items) of 20:1.^15^ To overcome the skewed distribution of high MMAS-8 scores in the sample and increase outcome validity the size of the target sample was increased by a factor of 2.5, resulting in a final sample size of 400 patients. During the study period 400 consecutive patients with hypertension who met the eligibility criteria for this study were selected at the clinics (Figure 1). The eligibility criteria were age older than 30 years, ability to communicate in the Korean language, receipt of a prescription for antihypertensive medication at the clinics during the 30 days before the study began, and no signs or symptoms of severe health problems such as cancer or chronic heart failure. During this period, 373 (93.3%) of the 400 patients with hypertension agreed to participate (Figure 1). The reasons for nonparticipation among the other 27 patients were insufficient time for participation (*n* = 22) and unwillingness to divulge personal information (*n* = 5). This study was approved by the Ethics Committee of Chung-Ang University Yong San Hospital and Chung-Ang University Hospital.

### Instrument and translation

The MMAS-8 was developed from a previously validated 4-item scale and supplemented with additional items regarding the context of adherence behavior.^15^ The theory underlying this measure is that failure to adhere to a medication regimen could be due to several factors, as expressed in the questions, “Do you sometimes forget to take your medication?”, “Do you stop taking medications when feeling worse?”, and “Do you feel hassled about sticking to a treatment plan?”. Each item measures a specific medication-taking behavior and not a determinant of adherence. Response categories are yes/no (dichotomous response) for items 1 through 7; item 8 uses a 5-point Likert scale. MMAS-8 scores range from 0 to 8 and have been trichotomized into 3 adherence levels to facilitate use in clinical practice, namely, high adherence (a score of 8), medium adherence (a score of ≥6 to <8), and low adherence (a score of <6). There are several reasons for dividing the score into 3 rather than 2 categories. First, numerous investigators have identified a linear positive correlation between MMAS-8 scale score and physiological response. Second, by dividing the scale into 3 categories, resources can be directed to patients having the greatest difficulty adhering to a treatment regimen, eg, by providing tailored educational counseling to address misconceptions regarding adherence. Finally, patients with high scores on the MMAS-8 can serve as models in focus group sessions, by detailing the processes by which they internalize adherence into their daily lifestyle.

For this study, hypertension was inserted into each item of the 8-item MMAS, which was then translated into Korean using forward and backward translation, using the recommendations of Wild et al for the translation and adaptation of patient-centered outcome measures.^16^ First, the original MMAS was forward-translated into the Korean by 2 qualified, independent language translators, both of whom were native speakers of Korean and proficient in English. Researchers reviewed the 2 primary versions and reached a consensus on a draft Korean version. Second, a bilingual Korean-Canadian expert translated the Korean draft back into English. Translators and researchers then compared the conceptual equivalence of the back-translated English version and the original. Third, the translated questionnaire was distributed to 30 Korean adults with hypertension who then completed the questionnaire and commented on the questions. These individuals were not included in the present study. Their comments were discussed by the researchers, after which a Korean final version was completed and made available for assessment of reliability and validity.

### Measurements

Face-to-face interviews were conducted to administer the MMAS-8 and collect data on sociodemographic characteristics and health behaviors such as smoking and alcohol consumption. At the same time, a calibrated mercury sphygmomanometer was used to measure BP twice, with a minimum interval of 5 minutes between measurements. All measurements were taken by a trained nurse according to guidelines established in the Seventh Report of the Joint National Committee.^17^ The patients’ medical records were reviewed to collect clinical information such as duration of hypertension, number of prescribed antihypertensive medications, and comorbidities. In addition, about one-third of the sample (*n* = 109) was randomly selected (using SPSS statistical software) to evaluate 2-week test-retest reliability via telephone. All patients who agreed to participate in telephone interviews were interviewed by the same people who conducted their baseline interviews.

### Statistical analysis

Sociodemographic characteristics, health behaviors, clinical characteristics, and MMAS-8 scores of the patients were assessed in relation to MMAS-8 category (high, moderate, low). The statistical significance of the characteristics and scores across the 3 adherence categories was determined using analysis of a variance (ANOVA) and χ^2^ tests for continuous variables and categorical variables, respectively. Potential ceiling and floor effects, which can affect reliability and validity, were considered if more than 15% of respondents achieved the lowest or highest possible total scores.^18^

Internal consistency of the 8-item scale was assessed using Cronbach α. Intraclass correlation (ICC) was used to assess test-retest reliability. According to Nunnally and Bernstein, newly developed measures can be accepted with a Cronbach α greater than 0.5; 0.7 should be the threshold in other cases.^19^ We considered an ICC less than 0.4 as poor, an ICC of 0.4 to 0.75 as fair or good, and an ICC greater than 0.75 as excellent.^20^

Convergent validity was evaluated using Pearson correlation coefficients between the MMAS-8 and the previous 4-item Morisky, Green, and Levine scale.^21^ Three items from the MMAS-8 are identical to items on the previous 4-item scale and were thus used to represent the previous scale. Known-groups validity was assessed using the association of MMAS-8 categories (high, moderate, low adherence) with BP control, as determined by the χ^2^ test. Additionally, multiple logistic regression was used to calculate odds ratios (ORs) for the association between MMAS-8 category and BP control, adjusted for age, sex, education, smoking, alcohol, obesity, duration of hypertension, number of antihypertensive drugs, and comorbidities.

Confirmatory factor analysis (CFA) and exploratory factor analysis (EFA)^22^ were used to examine the structural validity of the Korean version of the MMAS-8. First, CFA was used to evaluate fit of the scale in a 1-factor model, as in previous studies.^10^^,^^12^^–^^14^ The indices used to assess fit of the model were root mean square error of approximation (RMSEA), the Tucker-Lewis index (TLI) (also known as the non-normed fit index [NNFI]), and the comparative fit index (CFI). The goodness-of-fit criteria for each index were a TLI (NNFI) and CFI greater than 0.9 and an RMSEA less than 0.05.^23^^–^^25^ Second, EFA was used to identify factors unique to Korean patients with hypertension in the sample data. EFA with varimax rotation was used, and only factors with an eigenvalue greater than 1 were considered to contribute significantly to explaining variance. A factor loading greater than 0.3 on an item was considered to belong to the corresponding factor.^26^ All analyses were performed using IBM SPSS version 20.0 software with AMOS version 20.0. The level of significance was set at *P* less than 0.05.

## RESULTS

**Figure 2.**
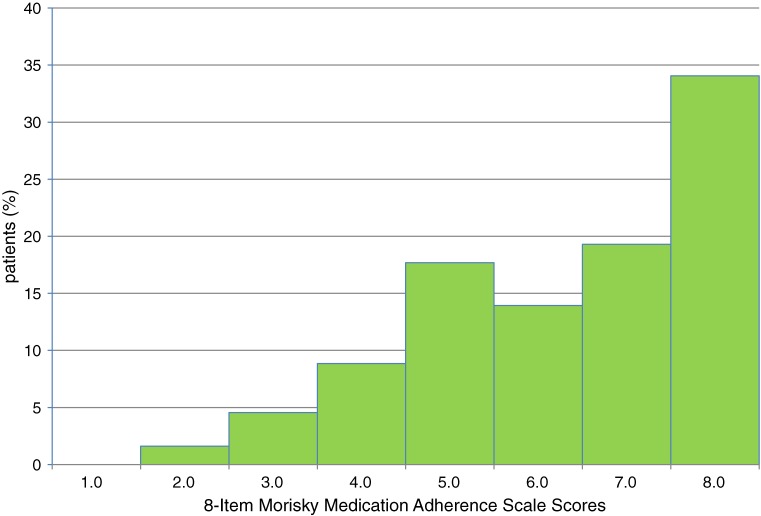
Distribution of scores on 8-item Morisky Medication Adherence Scale.

A total of 373 participants completed the study (Table 1). There were no significant differences among the 3 adherence groups with respect to sex, education level, number of comorbidities, or body mass index (BMI). However, there were significant differences in age, smoking status, frequency of alcohol drinking, duration of hypertension, systolic BP, diastolic BP, number of antihypertensive drugs, and MMAS-8 score. The distribution of MMAS scores was skewed (Figure 2), and the median was 7.0 (range, 2.5–8.0). A ceiling effect was observed, as almost one-third (34.0%) of respondents had a score of 8, ie, highest adherence to the hypertension treatment regimen. The distributions of responses to each item on the 8-item MMAS are shown in Table 2. The items with response rates greater than 50%, indicating good adherence, were not forgetting to take diabetes medications (63.8%) and having no days on which medication was not taken during the previous 2 weeks (64.9%). In contrast, more than 90% of respondents reported that they had taken their hypertension medications on the previous day and that they had not decided to stop or reduce their hypertension medications when they felt worse or better. In addition, most respondents never (59.0%) or rarely (35.4%) had difficulty remembering their medications.

**Table 1.** Characteristics of participants, by adherence category

Characteristic	Total Sample (*n* = 373)	High Adherence (MMAS = 8, *n* = 127)	Moderate Adherence (6 ≤ MMAS < 8, *n* = 124)	Low Adherence (MMAS < 6, *n* = 122)
Age, years^b^				
Mean (SD)	57.2 (11.20)	60.7 (10.49)	57.41 (11.2)	53.4 (10.8)
Range	30–83	30–82	32–83	30–82
Sex, %^a^				
Female	45.0	46.5	48.4	40.2
Male	55.0	53.5	51.6	59.8
Education, %^a^				
≤6th grade	13.4	12.5	16.9	10.7
7th–12th grade	45.8	52.8	41.9	42.6
College (2–4 years)	32.2	26.8	33.1	36.9
Graduate school	8.6	7.9	8.1	9.8
Body mass index^a^				
Mean (SD)	25.0 (3.2)	25.1 (3.1)	24.7 (3.1)	25.4 (3.1)
Range	17.9–39.6	17.9–39.6	18.3–38.2	18.4–34.4
Smoking status^b^				
Nonsmoker	59.4	63.8	66.1	47.9
Ex-smoker	23.9	22.0	19.4	30.6
Current smoker	16.7	14.2	14.5	21.5
Frequency of alcohol drinking^b^				
None	47.7	58.3	48.4	36.1
1/month	11.0	8.7	9.7	14.8
2–3/month	5.1	3.1	4.0	8.2
2–3/week	29.8	22.0	32.3	35.2
≥4/week	6.4	7.9	5.6	5.7
Duration of hypertension, months^b^				
Mean (SD)	52.75 (52.0)	62.2 (51.9)	54.8 (56.8)	40.9 (44.5)
Range	1.6–286.7	1.7–286.7	1.6–268.4	2.8–270.7
Systolic blood pressure, mm Hg^b^				
Mean (SD)	126.29 (10.4)	125.22 (8.3)	124.2 (8.9)	129.6 (12.9)
Range	99.0–189.0	110.0–150.0	102.5–150.0	99.0–189.0
Diastolic blood pressure, mm Hg^b^				
Mean (SD)	82.5 (6.7)	81.3 (4.9)	81.7 (6.2)	84.7 (8.1)
Range	67.5–120.5	68.8–98.0	68.5–99.5	67.5–120.5
No. of comorbidities, %^a^				
0	81.2	80.3	83.1	80.3
1	17.4	18.1	14.5	19.7
≥2	1.3	1.6	2.4	—
Antihypertensive drugs, *n*^b^				
Mean (SD)	2.2 (0.98)	2.4 (0.98)	2.2 (1.00)	2.02 (0.90)
Range	1–5	1–5	1–5	1–4
MMAS^b,c^				
Mean (SD)	6.6 (1.4)	8.0 (0.0)	6.8 (0.4)	5.0 (0.9)
Range	2.5–8.0	8.0–8.0	6.0–7.8	2.5–5.8

MMAS-8, 8-item Morisky Medication Adherence Scale.

^a^No significance difference among adherence groups (*P* ≥ 0.05).

^b^Significant difference among adherence groups (*P* < 0.05).

^c^Higher MMAS scores indicate greater adherence to medication.

**Table 2.** Distribution of responses to items on 8-item MMAS

Items 1–7				No *n* (%)	Yes *n* (%)
1. Do you sometimes forget to take your hypertension medications?				238 (63.8)	135 (36.2)
2. People sometimes miss taking their medications for reasons other than forgetting. Thinking over the past two weeks, were there any days when you did not take your hypertension medicine?				242 (64.9)	131 (35.1)
3. Have you ever cut back or stopped taking your medication without telling your doctor because you felt worse when you took it?				360 (96.5)	13 (3.5)
4. When you travel or leave home, do you sometimes forget to bring along your hypertension medications?				305 (81.8)	68 (18.2)
5. Did you take your hypertension medicine yesterday?				29 (7.8)	344 (92.2)
6. When you feel like your blood pressure is under control, do you sometimes stop taking your medicine?				346 (92.8)	27 (7.2)
7. Taking medication every day is a real inconvenience for some people. Do you ever feel hassled about sticking to your hypertension treatment regimen?				302 (81.0)	71 (19.0)

Item 8	Never *n* (%)	Rarely *n* (%)	Sometimes *n* (%)	Often *n* (%)	Always *n* (%)

8. How often do you have difficulty remembering to take all your hypertension medications?	220 (59.0)	132 (35.4)	19 (5.1)	2 (0.5)	0 (0.0)

MMAS, Morisky Medication Adherence Scale.

Use of the MMAS is protected by US copyright laws. Permission for use is required. A license agreement is available from: Donald E. Morisky, ScD, ScM, MSPH, Professor, Department of Community Health Sciences, UCLA Fielding School of Public Health, 650 Charles E. Young Drive South, Los Angeles, CA 90095-1772.

The Cronbach α for internal consistency was 0.56 for the MMAS-8 scale, which is below the generally accepted value of 0.70. However, the test-retest reliability of the MMAS-8 was excellent (ICC, 0.91; *P* < 0.001).

Analysis of convergent validity showed that the MMAS-8 was positively associated with the original 4-item Morisky, Green and Levine scale (*r* = 0.92; *P* < 0.01). The MMAS-8 had a good correlation with the 4-item Morisky scale.

Data on known-groups validity are shown in Tables 3 and 4. As shown in Table 3, the χ^2^ test showed a significant relationship between MMAS-8 adherence category and BP control (χ^2^ = 29.86, *P* < 0.001). As shown in Table 4, the prevalence of poor BP control (systolic BP ≥140 mm Hg or diastolic BP ≥90 mm Hg) in the low adherence group (MMAS-8 score <6) was about 5.1 times that among those with an MMAS-8 score of 6 or higher (adjusted OR, 5.08; 95% CI, 2.56–10.08).

**Table 3.** Relationship between MMAS-8 and blood pressure control

Parameter	Blood pressure control^a^		Total *n* (%)

	Poor control *n* (%)	Good control *n* (%)	
Low adherence (MMAS < 6)	36 (29.5)	86 (70.5)	122 (100)
Moderate adherence (6 ≤ MMAS < 8)	10 (8.1)	114 (91.9)	124 (100)
High adherence (MMAS = 8)	10 (7.9)	117 (92.1)	127 (100)
Total	56 (15.0)	317 (85.0)	373 (100)

MMAS-8, 8-item Morisky medication adherence scale.

^a^No. (%) of patients: χ^2^ = 29.855; *P* < 0.001.

**Table 4.** Odds ratios for poor blood pressure control associated with sociodemographic factors and adherence in univariate logistic regression and 2 multivariate logistic regression models

	Univariate analysis		Model I^c^		Model II^d^	
		
	OR^a^	95% CI^b^	OR^a^	95% CI^b^	OR	95% CI^a,b^
Age	0.962	0.937–0.987	0.975	0.938–1.013	0.981	0.943–1.021
Sex						
Male	Reference		Reference		Reference	
Female	0.528	0.290–0.960	0.617	0.228–1.668	0.636	0.237–1.712
Education						
≤6th grade	0.456	0.117–1.777	0.884	0.173–4.518	0.872	0.170–4.468
7th–12th grade	0.812	0.305–2.161	0.924	0.286–2.983	0.855	0.257–2.841
College (2–4 years)	0.815	0.296–2.247	0.710	0.224–2.252	0.687	0.213–2.217
Graduate school	Reference		Reference		Reference	
BMI						
Underweight	1.500	0.117–19.178	2.064	0.048–89.656	2.618	0.048–144.023
Normal	0.413	0.160–1.066	0.515	0.174–1.520	0.635	0.201–2.003
Overweight	0.629	0.238–1.660	0.608	0.207–1.791	0.738	0.238–2.288
Obese	Reference		Reference		Reference	
Smoking status						
Current smoker	2.727	1.348–5.518	1.396	0.524–3.722	1.489	0.552–4.013
Ex-smoker	1.463	0.722–2.966	0.611	0.215–1.738	0.654	0.227–1.886
Nonsmoker	Reference		Reference		Reference	
Frequency of alcohol drinking						
None						
1/month	Reference		Reference		Reference	
2–3/month	0.346	0.078–1.529	0.211	0.045–0.998	0.219	0.046–1.037
2–3/week	1.797	0.549–5.888	0.674	0.160–2.851	0.661	0.156–2.808
≥4/week	1.572	0.824–3.000	0.703	0.274–1.799	0.697	0.269–1.803
	2.246	0.808–6.245	1.256	0.336–4.691	1.167	0.302–4.515
Duration of hypertension (months)	0.995	0.988–1.001			0.995	0.987–1.004
No. of comorbidities						
0		Reference			Reference	
1	0.764	0.343–1.706			0.849	0.342–2.106
≥2	1.362	0.149–12.454			3.165	0.307–32.632
No. of antihypertensive drugs	1.157	0.868–1.542			1.304	0.919–1.850
Adherence group						
Low	4.835	2.653–8.810	4.890	2.529–9.456	5.075	2.555–10.079
Moderate/high		Reference		Reference		Reference

^a^Odds ratio.

^b^Confidence interval.

^c^Adjusted for age, sex, education, body mass index (BMI), smoking status, frequency of alcohol drinking.

^d^Adjusted for covariates in Model I plus duration of hypertension, number of comorbidities, and number of antihypertensive drugs.

Poor blood pressure control: systolic blood pressure ≥140 mm Hg or diastolic blood pressure ≥90 mm Hg.

The MMAS-8 showed low-to-moderate criterion-related validity, as shown in Table 3. With a cut-off point of 6 (low adherence, MMAS-8 score <6), the sensitivity, specificity, positive predictive value, and negative predictive value of the MMAS-8 were 64.3%, 72.9%, 29.5%, and 92.0% respectively. This sensitivity means that 64.3% of hypertensive patients who had poor BP control had low adherence (MMAS-8 score, <6), while the specificity indicates that 72.9% of patients with good BP control had moderate (MMAS-8 score, 6 to <8) or high (MMAS-8 score, 8) adherence to their medication. The positive predictive value indicates that 29.5% of the participants with low adherence had poorly controlled BP, whereas the negative predictive value means that 92.0% of those with moderate-to-high adherence had good BP control. When we changed the cut-off score for low adherence from 6 to 7 (low adherence, MMAS-8 score of <7), sensitivity, specificity, positive predictive value, and negative predictive value were 75.0%, 58.4%, 24.1%, and 93.0% respectively. Similarly, if the cut-off score was raised to 8 (low adherence, MMAS-8 score of <8), sensitivity, specificity, positive predictive value, and negative predictive value were 82.1%, 36.9%, 18.7%, and 92.1%, respectively.

CFA for the 1-factor model of the MMAS-8 showed a poor fit on the fit indices (RMSEA = 0.087, TLI = 0.825, and CFI = 0.875). As shown in Table 5, EFA showed 3 factors with eigenvalues greater than 1, which explained 58.5% of the total variance. Factor loadings between the 8 items of the MMAS and the 3 factors are presented. Factor 1 comprised items 1, 2, 4, 5, and 8, which mostly concern forgetting to take medications. Factor 2 consisted of items 3 and 6, which concern stopping medications when feeling better or worse. Factor 3 included only item 7, which concerns the view that taking medications daily is difficult.

**Table 5.** Exploratory factor analysis of the 8-Item Morisky Medication Adherence Scale in patients with hypertension^a^

Item	Factor 1	Factor 2	Factor 3
1	**0.799**	0.090	−0.143
2	**0.628**	0.111	0.159
3	0.032	**0.780**	0.037
4	**0.531**	−0.317	0.027
5	**0.413**	0.224	0.049
6	0.162	**0.783**	0.002
7	0.067	0.030	**0.982**
8	**0.862**	0.094	0.061

^a^Factor loading in 373 patients.

Bold-faced numbers indicate a factor loading >0.3.

## DISCUSSION

It is important to note that a ceiling effect was observed for the 8-item MMAS both in this study and in 2 previous studies.^11^^,^^14^ The consequences of a significant ceiling effect are that (1) changes in health behaviors cannot be measured in patients with the highest possible score and (2) false-negative case-findings (patients with the highest score on the scale and no BP control) are likely to be due to the limited number of items used to assess compliance behaviors.^18^^,^^27^ When observing changes in compliance with an antihypertensive medication regime, if response choices are added to the 7 items with binary responses (yes/no), score variability could improve among patients with the highest possible scores. Although specificity would be lower, this is considered an acceptable trade-off because sensitivity is more important than specificity in a clinical setting.

The Cronbach α for the MMAS-8 was below the acceptable level of 0.7 in previous studies^11^^–^^14^ (range, 0.54–0.67) and the present study (0.56). However, the low α in this study might be an underestimate of the internal consistency reliability of the scale, as 2 of the conditions required for Cronbach α to be an accurate estimate of reliability were not met.^28^^–^^31^ First, if α is to be a good estimate of reliability, then the measures involved should be unidimensional. Our EFA showed that the scale had 3 dimensions, ie, that there were 3 different traits in this scale. This could explain why α was lower than the acceptable level of 0.7. Moreover, if the number of test items is too small, α will underestimate the reliability of the scale.

Another explanation for the moderate Cronbach α is the low variability in the scale scores, which indicates that about half of the subjects in this study had values of 7 or 8, as shown in Figure 2. Internal consistency would be improved if variability among scale scores were greater, which would occur in a population with different levels of adherence.^32^ For instance, when the analysis was limited to patients with hypertension for less than 1 year (*n* = 74), Cronbach α increased to 0.60 because the variability in scale scores was greater among these patients. The proportion of those with a scale score greater than 7 was about 30%.

Finally, because 7 of the 8 items on the scale require binary responses (yes/no), which tends to decrease Cronbach α, internal consistency reliability could be improved by increasing the number of response choices.^32^ However, this was attempted on the Morisky, Green and Levine scale,^21^ with no change in internal consistency Nevertheless, the MMAS-8 had excellent test-retest reliability (ICC = 0.91, *P* < 0.001), indicating good stability of the scale over time. Excellent test-retest reliability has also been observed in other studies.^11^^–^^13^

Convergent validity was supported by the significant correlation with the previous 4-item scale (*r* = 0.92; *P* < 0.01), which was also shown in other studies.^11^^,^^12^ For known-groups validity, we found a significant association (χ^2^ = 29.86; *P* < 0.001) between MMAS-8 adherence category and BP control, as was noted in other studies.^11^^,^^12^ The adjusted OR for low adherence to poor BP control, which considered confounding variables for those associations, was 5.08 (95% CI, 2.56–10.08). These findings indicate that the scale could differentiate between patients with well and poorly controlled BP (systolic BP <140 mm Hg or diastolic BP <90 mm Hg).

However, using BP control as the gold standard, criterion-related validity was low or moderate, as was the case in previous studies.^11^^,^^12^ A possible reason for this finding is overestimation of adherence levels due to recall bias and social desirability. Adherence to medication can increase the number of clinic appointments, which might have a large effect on patient recall when the questionnaire was administered during a clinic appointment for hypertension treatment.^33^ Social desirability might have had an effect on question responses in the present study.^34^ Intentional nonadherence (eg, ceasing hypertension medication when feeling worse) was much less frequent than unintentional nonadherence (ie, forgetting to take hypertension medication), which suggests that patients could have answered questions in a way that increased MMAS scores even though their BP control was less than satisfactory. Increasing the cut-off score for low adherence from 6 to 8 would improve sensitivity, but at the expense of specificity. Nevertheless, this may be preferable because, in clinical practice, health care providers are more interested in identifying patients with both poor BP control and low adherence than in discovering those with good control and high adherence.

CFA of the unidimensional structure of the MMAS-8 in previous studies^10^^,^^12^^–^^14^ showed a poor fit in the present study. Construct validity analyzed by EFA with varimax rotation showed that the MMAS-8 had 3 factors with eigenvalues greater than 1: factor 1 (items 1, 2, 4, 5, and 8), factor 2 (items 3 and 6), and factor 3 (item 7). These results are similar to those reported in a study using a Thai version of the scale for patients with Type 2 diabetes.^11^ According to Morisky and colleagues,^10^ the MMAS-8 theoretically measures a specific medication-taking behavior that leads to failed adherence and not a determinant of adherence behavior. They indicated that this measurement could theoretically have more than 1 factor. Thus, it is unsurprising that the MMAS-8 showed 3 factors in this study and the study using the Thai version of the MMAS-8.^11^

This study had limitations. The respondents were recruited from outpatient clinics at 2 tertiary hospitals; therefore, the study sample was vulnerable to selection bias. Individuals with hypertension who are treated in outpatient clinics at tertiary hospitals must pay substantial out-of-pocket payments. Therefore, they are much more likely to adhere to their prescribed treatment, as compared with people with hypertension living in the community. In addition, MMAS scores may have been affected by social desirability and recall bias.

It may be necessary to modify the Korean MMAS-8 by increasing the number of response choices when examining changes in adherence after an intervention such as education.^35^ Nevertheless, the acceptable test-retest reliability and good convergent, known-groups, and construct validity indicate that the 8-item MMAS can be useful in assessing medication adherence among people with hypertension in a busy clinic in Korea. Moreover, it could help in identifying barriers to adherence and in developing targeted interventions to improve adherence by means of a “teachable moment” in a busy clinical setting. For example, when a physician identifies a patient with poor BP control who voluntarily stopped antihypertensive medication, the physician can immediately counsel the patient regarding the medication. Alternatively, for patients with an MMAS score of 8 and poor BP control, a change in therapy might be needed to achieve appropriate BP control. Our results may not be generalizable to other diseases. Thus, further research is needed in order to analyze the psychometric properties of the MMAS-8 in Koreans with other chronic conditions requiring long-term medical treatment, such as osteoporosis, ulcerative colitis, and tuberculosis.
